# Comparing the psychological benefits that Chinese and Western classical music preferences bring to cognitive emotion regulation, self-esteem, and psychological resilience

**DOI:** 10.3389/fpsyg.2025.1623200

**Published:** 2025-07-25

**Authors:** Xin Shan, Yuchen Wang, Long Luo

**Affiliations:** ^1^College of Music and Dance, China West Normal University, Nanchong, China; ^2^The Graduate School Arts and Culture, Sangmyung University, Seoul, Republic of Korea

**Keywords:** classical music, cognitive emotion regulation, self-esteem, psychological resilience, music preference

## Abstract

**Introduction:**

This study investigates the effects of preferences for Chinese and Western classical music on three key psychological constructs in adults: cognitive emotion regulation, self-esteem, and psychological resilience. Guided by the World Health Organization’s principles of mental health, the research aims to explore how preferences for these distinct musical traditions relate to individual psychological well-being.

**Methods:**

The study analyzed data from 604 valid responses collected through questionnaires, supplemented by behavioral observations, to examine the impact of classical music preferences on the targeted psychological outcomes.

**Results:**

Findings indicate that a preference for classical music (both Chinese and Western) significantly enhances cognitive emotion regulation, self-esteem, and psychological resilience, with the strongest effect observed on psychological resilience. Among moderating factors, age and educational attainment significantly influenced the relationship between music preference and the three psychological aspects, whereas gender had no significant moderating effect. Additionally, preference for Western classical music was found to be more effective than preference for Chinese classical music across all three dimensions, potentially attributed to its complex harmonic structure and rich emotional expression.

**Discussion:**

These results provide empirical evidence for the link between classical music preference and psychological well-being, offering new insights for the development of music therapy strategies and mental health interventions.

## 1 Introduction

The World Health Organization (WHO) emphasizes that “there is no health without mental health,” a principle that has achieved global consensus (Kuzman et al., 2020). In its in-depth interpretation of this grand principle, the academic community has widely acknowledged that positive mental health is not a monolithic state but is composed of several key capabilities. Among these, the ability to efficiently manage emotional fluctuations, known as cognitive emotion regulation (Garnefski et al., 2001; Saarikallio, 2011); a stable and positive perception of self-worth, or self-esteem (Rosenberg, 1965); and the capacity to adapt and recover from adversity, termed psychological resilience (Connor and Davidson, 2003), are recognized as its three indispensable pillars. These three components are intertwined, collectively forming a dynamic and complex system (Harvey and Boynton, 2021; Surzykiewicz et al., 2022).

Music, particularly classical music, is widely regarded as a tool for regulating psychological states, alleviating stress, and enhancing emotional expression (Rickard, 2004). Research indicates that music can evoke inner emotions, influence cognition, and shape behavioral responses (Koelsch, 2010). However, the psychological benefits of music are not uniform across all individuals and situations. The extent to which a piece of music can promote well-being often depends on an individual’s emotional connection to it. This naturally brings forth the crucial role of music preference, which acts as a key moderating factor between the musical stimulus and the psychological response. Music preference is not merely a transient opinion but a stable trait associated with personality and cognitive style (Rentfrow and Gosling, 2003). While some scholars define it as the degree of liking for a certain music genre (Rentfrow and Gosling, 2003), this study adopts a more experience-oriented definition: the degree of liking for a specific musical pattern after receiving the complete auditory stimulus, with its influence potentially persisting even after the listening experience has concluded. The congruence between a listener’s preference and the music is crucial for fostering positive emotional and cognitive responses (Saarikallio, 2011).

This study focuses on three core pillars of mental health relevant to the musical experience: cognitive emotion regulation, self-esteem, and psychological resilience. The selection of these variables is based on evidence from prior research revealing their specific connections with music. For instance, classical music, with its complex structures and harmonious melodies, provides a unique auditory environment for enhancing cognitive emotion regulation. It can evoke positive emotions while reducing negative ones (Chen, 2023), and individuals who prefer classical music often report using it as a strategy to manage their moods (Olsen et al., 2023). Secondly, music engagement and self-esteem are closely linked. Mastering musical skills, or even just deeply appreciating complex music, can cultivate a sense of accomplishment and self-worth (Culp, 2016). Finally, music is considered an important factor in building psychological resilience. For both children and adults, recreational music activities have been shown to enhance resilience by providing an outlet for stress and promoting adaptive coping mechanisms (Koehler et al., 2023; Tabibnia and Radecki, 2018).

Empirical research is a common method for exploring the multidimensional complexity of music and its effects (Li et al., 2024; Lin et al., 2025; Oncu, 2025; Oza and Behnaz, 2024; Togay and Firat, 2024). However, existing scholarship has disproportionately focused on the Western musical system (Fung, 2018; Hu et al., 2021). This scholarly gap becomes particularly salient when considering the stark differences in structure and cultural connotation between Chinese and Western classical music. Western classical music is known for its complex harmony and polyphonic textures, often guiding listeners through intense and cathartic emotional arcs. In contrast, Chinese classical music places greater emphasis on melody, timbre, and a connection with nature and philosophy, aiming to cultivate a meditative and introspective atmosphere (Thuc, 2018; Wang et al., 2021). These fundamental differences suggest they may yield distinct psychological benefits.

Therefore, this study aims to fill this gap by systematically analyzing the effects of preferences for Chinese and Western classical music on cognitive emotion regulation, self-esteem, and psychological resilience among Chinese adults. Employing a methodology of questionnaire surveys and behavioral observation, the core variables will be assessed through preference questions and standardized self-report scales. Simultaneously, potential confounding variables such as gender, age, and educational background will be carefully controlled to ensure the reliability and validity of the findings. This research is poised to provide new perspectives for music education and mental health interventions, with findings that could contribute to both theoretical foundations and practical applications, thereby offering valuable insights for policymakers, educators, and mental health professionals alike.

## 2 Theoretical background and assumptions

According to the SOR theoretical model established by Mehrabian and Russell (1974), individual behavioral responses essentially constitute a chained process whereby environmental stimuli induce psychological state alterations through neurocognitive mediation. This theoretical framework positions external environmental parameters as core independent variables that activate information processing within the central nervous system, with its operational pathway manifesting as sensory stimuli being transformed into psychological evaluation signals via thalamocortical pathways, subsequently triggering adaptive behavioral feedback (Drobesch-Binter, 2000). In interdisciplinary applications, this model demonstrates unique explanatory power in the field of music psychology: Juslin and Västfjäll (2008) revealed through psychoacoustic experiments how musical elements modulate emotional encoding in the limbic system, while Min et al. (2020) systematically demonstrated through ecological momentary assessment (EMA) methodology the reconstructive effects of musical environments on decision-making neural circuits. It is particularly noteworthy that as a cross-modal affective stimulus carrier, the congruence between musical structural features and individual cognitive schemata not only determines the formation of aesthetic preferences (Droe, 2006) but also profoundly engages in the functional construction of emotion regulation systems through neuroplasticity mechanisms. Therefore, we employ this theoretical framework to investigate the relationships between musical preferences and cognitive emotion regulation strategies, self-esteem levels, and psychological resilience.

Classical music has been widely recognized for its influence on cognitive emotion regulation, operating through a combination of physiological, cognitive, and social mechanisms. Research suggests that classical music activates the reward system, triggering endorphin release and reducing stress hormones like cortisol, thereby promoting relaxation and mood regulation (Peistaraite and Clark, 2020). From a cognitive perspective, its complex structures engage attention, fostering emotional reappraisal and memory associations that enhance positive affect. Socially, cultural exposure to classical traditions may reinforce emotional self-regulation through shared aesthetic experiences. In adulthood, musicians leverage these mechanisms to improve self-regulatory learning abilities, particularly through emotion regulation processes (López-íñiguez and Mcpherson, 2021). Even in distressing contexts, music facilitates brain re-engagement via neural pathways linked to reward processing and emotional control (Broughton et al., 2021). Studies on early 20th-century classical performances highlight how motor imagery and emotional observation during musical execution enhance cognitive reappraisal capacities (Pauwels et al., 2014). Therefore, we propose Hypothesis 1a (H1a): A preference for classical music (Chinese or Western) is closely associated with cognitive emotion regulation.

Self-esteem, defined as an individual’s overall assessment of self-worth, benefits from classical music through multiple pathways. Physiologically, compositions like Mozart’s reduce cortisol levels while stimulating endorphin production, improving mood and self-perception (Pauwels et al., 2014). Cognitively, music education fosters skill mastery and self-efficacy, with teachers using musical challenges to build student confidence. Socially, participation in musical communities creates identity reinforcement, where group engagement and cultural heritage enhance self-worth through shared achievements (Culp, 2016). Therefore, we propose Hypothesis 1b (H1b): A preference for classical music (Chinese or Western) is closely related to self-esteem.

Psychological resilience, an individual’s ability to adapt to stress, is also bolstered by classical music through interacting mechanisms. Physiologically, music regulates autonomic nervous system reactivity, improving heart rate variability and stress recovery (Kegelaers et al., 2021). Cognitively, musicians develop adaptive coping strategies by associating musical patterns with emotional control, enhancing stress tolerance. Socially, immersion in musical traditions provides cultural resources and peer support networks that buffer career-related pressures (Loveday et al., 2023). These combined effects have led to the use of classical music training to manage performance anxiety and enhance mental skills among students, particularly through metacognitive strategies that strengthen resilience (Osborne et al., 2014). Therefore, we propose Hypothesis 1c (H1c): A preference for classical music (Chinese or Western) is closely related to psychological resilience.

However, the psychological effects of music are not universal but are deeply embedded in cultural contexts. To explain how differences in musical structure lead to variations in psychological outcomes, this study draws upon the foundational framework of cultural self-construal theory (Markus and Kitayama, 1991). This theory posits that Western, individualistic cultures tend to foster an “independent self,” whose psychological needs focus on highlighting personal uniqueness, pursuing individual achievement, and directly expressing inner emotions. Conversely, East Asian, collectivistic cultures tend to cultivate an “interdependent self,” whose psychological needs emphasize maintaining interpersonal harmony, adapting to the environment, and achieving inner emotional peace.

This theoretical framework provides us with a key explanatory pathway. Western classical music, characterized by its complex harmony, polyphonic textures, and strong, dramatic “tension-resolution” structures, offers an effective avenue for the “independent self” to experience, process, and cathartically release strong emotions, thereby fulfilling its psychological needs for emotional intensity and self-affirmation (Weiß et al., 2019). In contrast, Chinese classical music is primarily based on monophonic melodies, emphasizing subtle changes in timbre, the linear flow of melody, and the artistic conception created by white space. This structure, which pursues introspection, subtlety, and balance, is highly aligned with the psychological tendency of the “interdependent self” to maintain inner tranquility and integrate into collective harmony (Thuc, 2018; Wang et al., 2021).

Based on the theoretical analysis above, we propose the following directional comparative hypotheses:

*Hypothesis 2a (H2a): Compared to Chinese classical music, Western classical music is more effective in enhancing cognitive emotion regulation.*

*Hypothesis 2b (H2b): Compared to Chinese classical music, Western classical music is more effective in enhancing self-esteem.*

*Hypothesis 2c (H2c): Compared to Chinese classical music, Western classical music is more effective in enhancing psychological resilience.*

## 3 Method

### 3.1 Data sources

In April 2024, in order to explore the psychological benefits of Chinese and Western classical music preferences in cognitive emotion regulation, self-esteem, and psychological resilience, we carried out a questionnaire survey among the Chinese public and conducted research combined with statistical analysis. This survey adopted a mixed-methods sampling strategy, with a total of 713 questionnaires distributed, including 108 paper questionnaires and 605 online questionnaires.

To enhance the diversity of the sample, our sampling was conducted through two main channels. Online, we utilized a professional sampling platform (“Wenjuanxing”) which pushed the questionnaire link to users in its national database based on its own stratified algorithms. Offline, our investigators employed a convenience sampling method in public places such as shopping malls, parks, and community centers across different regions of China, as well as at public transportation hubs like railway stations, to intercept and invite the public to participate. It is important to acknowledge that while this multi-pronged approach increases coverage, the non-probability nature of the convenience sampling component means the sample’s generalizability should be considered with care.

To encourage participants to actively complete the survey, we prepared random e-cash red envelopes ranging from 1 to 100 yuan for all respondents. We clearly informed the participants that their feedback would be used for the research on the impact of Chinese and Western classical music on psychological well-being, and that all data would be kept strictly confidential and used for academic research only. Eventually, a total of 713 questionnaires were recovered, with 604 valid questionnaires, and the effective recovery rate reached 84.71%. This high response rate ensures the reliability of the collected data for this specific sample, providing a solid foundation for subsequent analysis.

To address the critical importance of a shared cultural background, all participants in this study were citizens of the People’s Republic of China. This ensures a consistent national context for interpreting their psychological responses to native and foreign musical traditions.

Furthermore, to address potential confounding effects and enhance the validity of our conclusions, we collected detailed demographic data from all participants. These variables, particularly those related to socioeconomic status (e.g., education, income) and cultural background (e.g., urban/rural residence, ethnicity), were systematically recorded. The express purpose of collecting this information was to allow for their inclusion as control variables in the subsequent statistical analyses. This methodological step enables a more precise examination of the relationship between music preference and psychological well-being by statistically accounting for the influence of these key demographic factors.

The demographic profile of the 604 valid respondents, used for both descriptive and control purposes, is as follows: The age distribution was 5.13% under 18, 27.82% from 18–25, 30.30% from 26–35, 19.54% from 36–45, 11.09% from 46–55, and 6.13% over 56. In terms of gender, 63.08% were male and 36.92% were female. For education level, 9.27% had a junior high school education or below, 21.19% had a high school education, 19.54% had a college education, 39.74% held a bachelor’s degree, and 10.27% held a master’s degree or higher.

Regarding marital status, 69.37% were unmarried, and 30.63% were married. For residence, 62.42% lived in urban areas, and 37.58% in rural areas. In terms of annual income, 20.20% earned less than 50,000 yuan, 38.41% earned 50,000–100,000 yuan, 28.97% earned 100,000–200,000 yuan, 8.44% earned 200,000–300,000 yuan, and 3.97% earned more than 300,000 yuan. The ethnic distribution showed that 76.16% were Han Chinese, while 23.84% belonged to ethnic minorities. For clarity, the term “ethnic minorities” in the context of China refers to the 55 officially recognized non-Han ethnic groups. Including this demographic reflects the country’s multi-ethnic composition. Finally, in terms of religious affiliation, 27.82% identified with a religious belief, while 72.18% did not.

### 3.2 Ethics approval

The preliminary survey of this study, titled “Comparing the Psychological Benefits that Chinese and Western Classical Music Preferences Bring to Cognitive Emotion Regulation, Self-Esteem, and Psychological Resilience” involved the distribution of 713 questionnaires. Informed consent was obtained from all participants. The study was approved by the Institutional Review Board (IRB) of Sangmyung University. In line with national regulations, the study was deemed exempt from full ethics review as it was non-interventional and observational. The research adhered to the ethical principles outlined in the Declaration of Helsinki, ensuring the protection of participants’ rights and confidentiality.

### 3.3 Definition of variables

The questionnaire was structured into six dimensions: basic information, Western classical music preference, Chinese classical music preference, emotion regulation, self-esteem, and psychological resilience.

The first dimension collected participants’ demographic information, including age, sex, education level, marital status, area of residence, annual income, ethnicity, and religion. The second dimension assessed participants’ preferences for Western classical music, including chamber music, symphonic music, and foreign piano works, referencing Larson’s (1995) study (Larson, 1995; Liu et al., 2021). The third dimension examined preferences for Chinese classical music, including Chinese folk songs, Chinese folk music, and Chinese opera, also referencing Liu and Larson’s study. This assessment tool comprises 6 measurement dimensions employing a standardized 5-point Likert scale (1 = strongly disagree, 2 = disagree, 3 = neutral, 4 = agree, 5 = strongly agree; Table 1).

**Table 1:** Statistics on the composition of the valid samples.

Name (of a thing)	Options (as in computer software settings)	Frequency	Percentage (%)	Cumulative percentage (%)
Your age	Under 18	31	5.132	5.132
	18–25 years	168	27.815	32.947
	26–35 years	183	30.298	63.245
	36–45 years	118	19.536	82.781
	46–55 years	67	11.093	93.874
	56+	37	6.126	100.000
Gender	Male	381	63.079	63.079
	Women	223	36.921	100.000
Level of education	Junior high school and below	56	9.272	9.272
	With HS education	128	21.192	30.464
	Three-year college	118	19.536	50.000
	Undergraduate (adjective)	240	39.735	89.735
	Master’s degree or above	62	10.265	100.000
Marital status	Unmarried	419	69.371	69.371
	Married	185	30.629	100.000
Area of residence	Municipalities	377	62.417	62.417
	Countryside	227	37.583	100.000
Annual income (in RMB)	Less than $50,000	122	20.199	20.199
	$50,000–$100,000	232	38.411	58.609
	$100,000–$200,000	175	28.974	87.583
	$200,000–$300,000	51	8.444	96.026
	More than $300,000	24	3.974	100.000
Your ethnicity is	Han ethnic group	460	76.159	76.159
	Non-Han	144	23.841	100.000
Religious	With Religious Belief	168	27.815	27.815
	Without Religious Belief	436	72.185	100.000
Total		604	100.000	100.000

The fourth dimension focused on emotion regulation, measuring participants’ strategies for managing emotions, such as self-attribution, acceptance of past events, and situational acceptance, based on Garnefski et al. (2001). The fifth dimension evaluated self-esteem, assessing aspects such as satisfaction with oneself and perception of worthlessness, following Rosenberg’s (1965) self-esteem scale. The sixth dimension investigated psychological resilience, including adaptability to change and intimacy, using the Connor-Davidson Resilience Scale (Connor and Davidson, 2003). For a detailed description of the variables, refer to Table 2.

**Table 2:** Variable definitions and assignments.

Typology	Variable name	Encodings	Variable assignment
Basics	(a person’s) age	age	1 = Under 18, 2 = 18–25, 3 = 26–35, 4 = 36–45, 5 = 46–55, 6 = 56 and over
	distinguishing between the sexes	gnd	1 = Male, 2 = Female
	educational level	edu	1 = Middle school and below, 2 = High school, 3 = College, 4 = Bachelor’s degree, 5 = Master’s degree and above
	marital status	mrtl	1 = Unmarried, 2 = Married
	Area of residence	area	1 = Urban, 2 = Rural
	Annual income (RMB)	incm	1 = less than $50,000, 2 = $50,000–$100,000, 3 = $100,000–$200,000, 4 = $200,000–$300,000, 5 = more than $300,000
	ethnic group	ethn	1 = Han, 2 = Non-Han
	religious belief	relg	1 = Yes, 2 = No
Western Classical Music Preferences (wcmp)	I like chamber music.	wcmp_1	1 = very dislike, 2 = dislike, 3 = fairly, 4 = like, 5 = very like
	Love the symphony.	wcmp_2	1 = very dislike, 2 = dislike, 3 = fairly, 4 = like, 5 = very like
	Love foreign piano works	wcmp_3	1 = very dislike, 2 = dislike, 3 = fairly, 4 = like, 5 = very like
Classical Chinese Music Preferences (ccmp)	Love Chinese folk songs	ccmp_1	1 = Very disliked, 2 = Disliked, 3 = Average, 4 = Liked, 5 = Very liked
	Love Chinese folk music	ccmp_2	1 = Very disliked, 2 = Disliked, 3 = Average, 4 = Liked, 5 = Very liked
	Loves Chinese opera and song	ccmp_3	1 = Very disliked, 2 = Disliked, 3 = Average, 4 = Liked, 5 = Very liked
Emotional regulation (emo)	Feeling like I’m to blame.	emo_1	5 = Never,4 = Almost never, 3 = Sometimes, 2 = Almost always, 1 = Always
	Feeling like I’m the responsible one	emo_2	5 = Never,4 = Almost never, 3 = Sometimes, 2 = Almost always, 1 = Always
	I made the mistake.	emo_3	5 = Never,4 = Almost never, 3 = Sometimes, 2 = Almost always, 1 = Always
	The reason for this is me.	emo_4	5 = Never,4 = Almost never, 3 = Sometimes, 2 = Almost always, 1 = Always
	You have to accept what happened.	emo_5	1 = Never, 2 = Almost never, 3 = Sometimes, 4 = Almost always, 5 = Always
	Acceptable status	emo_6	1 = Never, 2 = Almost never, 3 = Sometimes, 4 = Almost always, 5 = Always
	It does not change anything.	emo_7	1 = Never, 2 = Almost never, 3 = Sometimes, 4 = Almost always, 5 = Always
	One must learn to accept it.	emo_8	1 = Never, 2 = Almost never, 3 = Sometimes, 4 = Almost always, 5 = Always
	Recall the feeling of the experience	emo_9	5 = Never,4 = Almost never, 3 = Sometimes, 2 = Almost always, 1 = Always
	Addicted to the feeling of experiencing	emo_10	5 = Never,4 = Almost never, 3 = Sometimes, 2 = Almost always, 1 = Always
	Understand the reason for the feeling	emo_11	5 = Never,4 = Almost never, 3 = Sometimes, 2 = Almost always, 1 = Always
	Think about the feelings evoked	emo_12	5 = Never,4 = Almost never, 3 = Sometimes, 2 = Almost always, 1 = Always
	Think of something better.	emo_13	1 = Never, 2 = Almost never, 3 = Sometimes, 4 = Almost always, 5 = Always
	Think of something pleasant.	emo_14	1 = Never, 2 = Almost never, 3 = Sometimes, 4 = Almost always, 5 = Always
	think of something good	emo_15	1 = Never, 2 = Almost never, 3 = Sometimes, 4 = Almost always, 5 = Always
	Think of a pleasant experience.	emo_16	1 = Never, 2 = Almost never, 3 = Sometimes, 4 = Almost always, 5 = Always
	Trying to do what’s best.	emo_17	1 = Never, 2 = Almost never, 3 = Sometimes, 4 = Almost always, 5 = Always
	How do you want to deal with the situation?	emo_18	1 = Never, 2 = Almost never, 3 = Sometimes, 4 = Almost always, 5 = Always
	How do you want to change the situation?	emo_19	1 = Never, 2 = Almost never, 3 = Sometimes, 4 = Almost always, 5 = Always
	Trying to make the best plan	emo_20	1 = Never, 2 = Almost never, 3 = Sometimes, 4 = Almost always, 5 = Always
	Learned something.	emo_21	1 = Never, 2 = Almost never, 3 = Sometimes, 4 = Almost always, 5 = Always
	Things that make me stronger	emo_22	1 = Never, 2 = Almost never, 3 = Sometimes, 4 = Almost always, 5 = Always
	Cognitive Emotion Regulation Strategies	emo_23	5 = Never,4 = Almost never, 3 = Sometimes, 2 = Almost always, 1 = Always
	Finding Cognitive Emotion Regulation Strategies	emo_24	1 = Never, 2 = Almost never, 3 = Sometimes, 4 = Almost always, 5 = Always
	Thinking everything’s going to go bad.	emo_25	1 = Never, 2 = Almost never, 3 = Sometimes, 4 = Almost always, 5 = Always
	Wanting someone to have a worse experience.	emo_26	1 = Never, 2 = Almost never, 3 = Sometimes, 4 = Almost always, 5 = Always
	Guess it was not so bad.	emo_27	1 = Never, 2 = Almost never, 3 = Sometimes, 4 = Almost always, 5 = Always
	I think there are worse things in life.	emo_28	1 = Never, 2 = Almost never, 3 = Sometimes, 4 = Almost always, 5 = Always
	Think I have been through worse than anyone else.	emo_29	5 = Never,4 = Almost never, 3 = Sometimes, 2 = Almost always, 1 = Always
	It is scary to keep wanting to go through	emo_30	5 = Never,4 = Almost never, 3 = Sometimes, 2 = Almost always, 1 = Always
	Trying to experience the worst.	emo_31	5 = Never,4 = Almost never, 3 = Sometimes, 2 = Almost always, 1 = Always
	It is scary to keep thinking about things.	emo_32	1 = Never, 2 = Almost never, 3 = Sometimes, 4 = Almost always, 5 = Always
	Feeling that others are to blame	emo_33	5 = Never,4 = Almost never, 3 = Sometimes, 2 = Almost always, 1 = Always
	Feeling that others are responsible for what happens	emo_34	5 = Never,4 = Almost never, 3 = Sometimes, 2 = Almost always, 1 = Always
	Mistakes are made by other people.	emo_35	5 = Never,4 = Almost never, 3 = Sometimes, 2 = Almost always, 1 = Always
	The reason for this is someone else’s.	emo_36	5 = Never,4 = Almost never, 3 = Sometimes, 2 = Almost always, 1 = Always
Self-confidence (esteem)	Overall, I’m happy with myself	esteem_1	1 = Disagree completely, 2 = Disagree, 3 = Agree, 4 = Agree completely
	Sometimes I feel worthless.	esteem_2	4 = Disagree completely, 3 = Disagree, 2 = Agree, 1 = Agree completely
	I think I have some good qualities.	esteem_3	1 = Disagree completely, 2 = Disagree, 3 = Agree, 4 = Agree completely
	I’m capable of doing things as well as most people.	esteem_4	1 = Disagree completely, 2 = Disagree, 3 = Agree, 4 = Agree completely
	I do not feel like I have anything in me to be proud of.	esteem_5	4 = Disagree completely, 3 = Disagree, 2 = Agree, 1 = Agree completely
	Sometimes I feel useless.	esteem_6	4 = Disagree completely, 3 = Disagree, 2 = Agree, 1 = Agree completely
	I feel like I’m a valuable person, at least as much as anyone else	esteem_7	1 = Disagree completely, 2 = Disagree, 3 = Agree, 4 = Agree completely
	I’d like to be able to think more highly of myself.	esteem_8	4 = Disagree completely, 3 = Disagree, 2 = Agree, 1 = Agree completely
	Across the board, I tend to think of myself as a loser	esteem_9	4 = Disagree completely, 3 = Disagree, 2 = Agree, 1 = Agree completely
	I have a positive outlook on myself.	esteem_10	1 = Disagree completely, 2 = Disagree, 3 = Agree, 4 = Agree completely
Mental toughness (resilience)	I can adapt to change.	resilience_1	1 = never, 2 = rarely, 3 = sometimes, 4 = often, 5 = always
	I have close, secure relationships	resilience_2	1 = never, 2 = rarely, 3 = sometimes, 4 = often, 5 = always
	Sometimes fate or God can help.	resilience_3	1 = never, 2 = rarely, 3 = sometimes, 4 = often, 5 = always
	I can handle whatever happens.	resilience_4	1 = never, 2 = rarely, 3 = sometimes, 4 = often, 5 = always
	My past successes have given me the confidence to face challenges	resilience_5	1 = never, 2 = rarely, 3 = sometimes, 4 = often, 5 = always
	I can see the humor in things.	resilience_6	1 = never, 2 = rarely, 3 = sometimes, 4 = often, 5 = always
	Coping with stress makes me feel empowered	resilience_7	1 = never, 2 = rarely, 3 = sometimes, 4 = often, 5 = always
	After a hardship or illness, I tend to recover quickly	resilience_8	1 = never, 2 = rarely, 3 = sometimes, 4 = often, 5 = always
	Things happen for a reason.	resilience_9	1 = never, 2 = rarely, 3 = sometimes, 4 = often, 5 = always
	Whatever the outcome, I will do my best.	resilience_10	1 = never, 2 = rarely, 3 = sometimes, 4 = often, 5 = always
	I can achieve my goals.	resilience_11	1 = never, 2 = rarely, 3 = sometimes, 4 = often, 5 = always
	I do not give up easily when things do not look promising.	resilience_12	1 = never, 2 = rarely, 3 = sometimes, 4 = often, 5 = always
	I know where to go for help.	resilience_13	1 = never, 2 = rarely, 3 = sometimes, 4 = often, 5 = always
	I am able to concentrate and think clearly under pressure	resilience_14	1 = never, 2 = rarely, 3 = sometimes, 4 = often, 5 = always
	I like to take the lead in problem solving	resilience_15	1 = never, 2 = rarely, 3 = sometimes, 4 = often, 5 = always
	I will not be discouraged by failure.	resilience_16	1 = never, 2 = rarely, 3 = sometimes, 4 = often, 5 = always
	I consider myself a powerful man.	resilience_17	1 = never, 2 = rarely, 3 = sometimes, 4 = often, 5 = always
	I can make unusual or difficult decisions.	resilience_18	1 = never, 2 = rarely, 3 = sometimes, 4 = often, 5 = always
	I can handle unhappiness.	resilience_19	1 = never, 2 = rarely, 3 = sometimes, 4 = often, 5 = always
	I had to act on a hunch.	resilience_20	1 = never, 2 = rarely, 3 = sometimes, 4 = often, 5 = always
	I have a strong sense of purpose.	resilience_21	1 = never, 2 = rarely, 3 = sometimes, 4 = often, 5 = always
	I feel in control of my life.	resilience_22	1 = never, 2 = rarely, 3 = sometimes, 4 = often, 5 = always
	I like a challenge.	resilience_23	1 = never, 2 = rarely, 3 = sometimes, 4 = often, 5 = always
	I worked hard to get there.	resilience_24	1 = never, 2 = rarely, 3 = sometimes, 4 = often, 5 = always
	I’m proud of my accomplishments.	resilience_25	1 = never, 2 = rarely, 3 = sometimes, 4 = often, 5 = always

## 4 Empirical results and analysis

### 4.1 Validity testing of questionnaires

#### 4.1.1 Normal distribution test

A fundamental assumption for statistical analysis of questionnaire data is that the data follow a normal distribution. To assess this, we examined the mean, standard deviation, skewness, and kurtosis of the questionnaire items. It is generally accepted that when the absolute value of the skewness coefficient is less than 3 and the absolute value of the kurtosis coefficient is less than 8, the data can be considered normally distributed (Park, 2021). The specific test results are shown in Table 3.

**Table 3:** Descriptive statistics for each item.

variable name	Mean	Standard deviation (SD)	Skewness	kurtosis
wcmp_1	3.447	1.301	−0.367	1.923
wcmp_2	3.450	1.300	−0.387	1.943
wcmp_3	3.483	1.315	−0.393	1.905
ccmp_1	3.411	1.262	−0.301	1.966
ccmp_2	3.394	1.253	−0.248	1.931
ccmp_3	3.387	1.327	−0.339	1.890
emo_1	3.068	1.266	−0.275	1.973
emo_2	3.088	1.255	−0.236	2.024
emo_3	3.162	1.352	−0.288	1.885
emo_4	3.111	1.304	−0.336	1.953
emo_5	3.671	1.278	−0.554	2.074
emo_6	3.579	1.272	−0.527	2.133
emo_7	3.561	1.262	−0.529	2.180
emo_8	3.637	1.252	−0.574	2.215
emo_9	3.127	1.260	−0.276	1.993
emo_10	3.094	1.268	−0.265	1.983
emo_11	3.091	1.258	−0.287	1.986
emo_12	3.088	1.283	−0.244	1.968
emo_13	3.619	1.275	−0.563	2.131
emo_14	3.611	1.235	−0.462	2.044
emo_15	3.581	1.235	−0.547	2.194
emo_16	3.553	1.276	−0.550	2.147
emo_17	3.596	1.252	−0.518	2.117
emo_18	3.576	1.281	−0.532	2.093
emo_19	3.601	1.256	−0.574	2.191
emo_20	3.649	1.242	−0.540	2.137
emo_21	3.541	1.288	−0.495	2.060
emo_22	3.594	1.265	−0.537	2.101
emo_23	3.109	1.282	−0.266	1.968
emo_24	3.606	1.259	−0.494	2.045
emo_25	3.556	1.287	−0.524	2.086
emo_26	3.621	1.257	−0.533	2.067
emo_27	3.576	1.264	−0.502	2.091
emo_28	3.560	1.252	−0.516	2.122
emo_29	3.036	1.248	−0.248	2.007
emo_30	3.093	1.286	−0.295	1.947
emo_31	3.131	1.311	−0.291	1.919
emo_32	3.593	1.290	−0.555	2.142
emo_33	3.113	1.259	−0.307	2.006
emo_34	3.091	1.290	−0.230	1.955
emo_35	3.113	1.275	−0.240	2.001
emo_36	3.089	1.279	−0.324	1.978
esteem_1	2.748	1.001	−0.395	2.111
esteem_2	2.740	0.974	−0.388	2.187
esteem_3	2.760	0.983	−0.387	2.155
esteem_4	2.738	1.026	−0.401	2.044
esteem_5	2.783	0.993	−0.390	2.116
esteem_6	2.770	0.993	−0.391	2.122
esteem_7	2.753	0.981	−0.388	2.162
esteem_8	2.791	0.986	−0.385	2.128
esteem_9	2.795	0.985	−0.384	2.128
esteem_10	2.772	1.011	−0.399	2.076
resilience_1	3.654	1.233	−0.516	2.076
resilience_2	3.606	1.280	−0.540	2.060
resilience_3	3.619	1.267	−0.565	2.147
resilience_4	3.619	1.259	−0.567	2.179
resilience_5	3.598	1.244	−0.508	2.147
resilience_6	3.639	1.255	−0.551	2.116
resilience_7	3.601	1.264	−0.586	2.210
resilience_8	3.616	1.252	−0.561	2.188
resilience_9	3.568	1.263	−0.536	2.155
resilience_10	3.579	1.261	−0.532	2.145
resilience_11	3.614	1.276	−0.570	2.157
resilience_12	3.639	1.264	−0.576	2.171
resilience_13	3.621	1.283	−0.536	2.079
resilience_14	3.634	1.262	−0.576	2.127
resilience_15	3.623	1.263	−0.544	2.144
resilience_16	3.618	1.280	−0.630	2.253
resilience_17	3.566	1.288	−0.538	2.113
resilience_18	3.601	1.284	−0.575	2.152
resilience_19	3.614	1.240	−0.571	2.221
resilience_20	3.575	1.268	−0.526	2.095
resilience_21	3.631	1.254	−0.560	2.178
resilience_22	3.644	1.250	−0.604	2.277
resilience_23	3.632	1.284	−0.537	2.057
resilience_24	3.624	1.243	−0.541	2.158
resilience_25	3.629	1.277	−0.550	2.086

Table 3 shows that the absolute values of the skewness coefficients are all below 1, and the absolute values of the kurtosis coefficients are all less than 2. These results indicate that the data conform to a normal distribution.

#### 4.1.2 Reliability analysis

To assess data reliability, Cronbach’s alpha test was conducted using SPSS 20.0. The results, presented in Table 4, indicate that the Cronbach’s alpha coefficients for each scale are greater than 0.8, demonstrating a high level of reliability. This suggests that the collected data are internally consistent and reliable.

**Table 4:** Cronbach’s alpha coefficients for the scale.

Variant	Quantities	Cronbach’s alpha coefficient	Confidence level
wcmp	3	0.901	High reliability
ccmp	3	0.884	High reliability
emo	36	0.990	High reliability
esteem	10	0.969	High reliability
resilience	25	0.986	High reliability

#### 4.1.3 Validity analysis

Structural validity was assessed using the KMO test, Bartlett’s spherical test, cumulative contribution rate, and factor loading analysis. The KMO test and Bartlett’s spherical test were conducted to determine whether the questionnaire data were suitable for factor analysis. The cumulative contribution rate represents the extent to which common factors explain variance, while factor loading measures the correlation between the original variables and the extracted common factor.

The analysis results show that the KMO value is 0.993, and Bartlett’s test of sphericity yields an approximate chi-square value of 54243.08 (*p* < 0.001), confirming that the data are appropriate for factor analysis. As shown in Table 5, five principal components were extracted using an eigenvalue threshold of greater than 1, with a cumulative explained variance of 75.39%. All 73 items aligned with the original factor structure, and no cross-factor loadings were observed. These findings indicate that the scale exhibits relatively strong structural validity.

**Table 5:** Exploratory factor analysis of the scale.

Table of factor loading coefficients after rotation						Commonality (common factor variance)
	Postrotation factor loading coefficients					
	Factor 1	Factor 2	Factor 3	Factor 4	Factor 5	
wcmp_1	0.409	0.386	0.256	0.105	0.66	0.829
wcmp_2	0.352	0.421	0.274	0.075	0.665	0.824
wcmp_3	0.376	0.402	0.243	0.091	0.685	0.84
ccmp_1	0.292	0.283	0.207	0.779	0.057	0.817
ccmp_2	0.334	0.295	0.183	0.755	0.074	0.808
ccmp_3	0.323	0.343	0.234	0.727	0.068	0.81
emo_1	0.734	0.370	0.206	0.107	0.094	0.739
emo_2	0.753	0.304	0.195	0.068	0.133	0.719
emo_3	0.748	0.326	0.220	0.104	0.067	0.729
emo_4	0.735	0.343	0.190	0.138	0.165	0.741
emo_5	0.729	0.355	0.254	0.115	0.142	0.755
emo_6	0.760	0.316	0.195	0.084	0.116	0.735
emo_7	0.721	0.348	0.237	0.119	0.094	0.720
emo_8	0.738	0.353	0.209	0.104	0.095	0.733
emo_9	0.745	0.344	0.216	0.082	0.051	0.730
emo_10	0.742	0.310	0.234	0.153	0.070	0.730
emo_11	0.734	0.344	0.227	0.133	0.079	0.732
emo_12	0.741	0.327	0.228	0.069	0.090	0.721
emo_13	0.770	0.302	0.228	0.102	0.113	0.760
emo_14	0.742	0.348	0.200	0.115	0.048	0.727
emo_15	0.730	0.379	0.209	0.095	0.130	0.745
emo_16	0.751	0.317	0.258	0.105	0.115	0.756
emo_17	0.737	0.325	0.234	0.133	0.134	0.739
emo_18	0.752	0.321	0.218	0.133	0.131	0.752
emo_19	0.747	0.334	0.240	0.126	0.105	0.755
emo_20	0.729	0.364	0.264	0.116	0.047	0.749
emo_21	0.763	0.321	0.193	0.108	0.101	0.744
emo_22	0.762	0.311	0.252	0.125	0.090	0.764
emo_23	0.740	0.335	0.219	0.127	0.047	0.726
emo_24	0.755	0.334	0.208	0.103	0.087	0.744
emo_25	0.746	0.371	0.169	0.125	0.111	0.750
emo_26	0.751	0.335	0.256	0.118	0.135	0.773
emo_27	0.749	0.310	0.242	0.113	0.069	0.733
emo_28	0.752	0.357	0.226	0.088	0.045	0.753
emo_29	0.729	0.339	0.224	0.111	0.120	0.724
emo_30	0.724	0.339	0.265	0.126	0.128	0.741
emo_31	0.745	0.336	0.236	0.132	0.059	0.745
emo_32	0.731	0.324	0.237	0.144	0.137	0.734
emo_33	0.732	0.360	0.204	0.097	0.094	0.726
emo_34	0.767	0.265	0.199	0.123	0.100	0.723
emo_35	0.725	0.324	0.261	0.092	0.073	0.713
emo_36	0.754	0.304	0.216	0.105	0.125	0.734
esteem_1	0.353	0.370	0.715	0.111	0.07	0.789
esteem_2	0.377	0.347	0.696	0.153	0.137	0.79
esteem_3	0.353	0.386	0.697	0.118	0.110	0.785
esteem_4	0.390	0.374	0.672	0.159	0.124	0.784
esteem_5	0.348	0.391	0.696	0.135	0.054	0.780
esteem_6	0.392	0.385	0.672	0.128	0.098	0.779
esteem_7	0.352	0.371	0.701	0.084	0.108	0.772
esteem_8	0.395	0.371	0.683	0.104	0.088	0.779
esteem_9	0.368	0.411	0.661	0.084	0.194	0.786
esteem_10	0.385	0.392	0.668	0.141	0.101	0.778
resilience_1	0.346	0.742	0.251	0.084	0.111	0.753
resilience_2	0.379	0.723	0.248	0.135	0.133	0.764
resilience_3	0.374	0.729	0.243	0.106	0.091	0.750
resilience_4	0.329	0.766	0.183	0.126	0.099	0.755
resilience_5	0.324	0.729	0.224	0.144	0.124	0.724
resilience_6	0.377	0.734	0.235	0.111	0.116	0.761
resilience_7	0.370	0.733	0.221	0.108	0.097	0.744
resilience_8	0.339	0.744	0.220	0.125	0.087	0.740
resilience_9	0.347	0.736	0.228	0.085	0.141	0.741
resilience_10	0.378	0.724	0.218	0.100	0.084	0.732
resilience_11	0.356	0.755	0.193	0.096	0.103	0.754
resilience_12	0.340	0.747	0.222	0.135	0.092	0.749
resilience_13	0.364	0.729	0.247	0.107	0.122	0.751
resilience_14	0.387	0.740	0.211	0.108	0.131	0.770
resilience_15	0.362	0.720	0.242	0.125	0.068	0.729
resilience_16	0.382	0.716	0.285	0.090	0.091	0.757
resilience_17	0.333	0.750	0.248	0.114	0.042	0.750
resilience_18	0.353	0.742	0.227	0.136	0.114	0.758
resilience_19	0.346	0.742	0.243	0.104	0.067	0.746
resilience_20	0.372	0.717	0.268	0.133	0.110	0.754
resilience_21	0.387	0.722	0.224	0.107	0.093	0.741
resilience_22	0.332	0.726	0.256	0.129	0.123	0.734
resilience_23	0.347	0.734	0.255	0.104	0.113	0.749
resilience_24	0.322	0.736	0.241	0.144	0.107	0.736
resilience_25	0.379	0.745	0.226	0.084	0.080	0.762
Cumulative proportion of variance explained	32.742%	58.396%	69.116%	72.6%	75.386%	
KMO value	0.993					
Bartlett’s test of sphericity	Approximate Chi-square (math.)			54243.08		
	P			0.000***		

### 4.2 Correlation analysis

This study examines the effects of Chinese and Western classical music preferences on individuals’ cognitive emotion regulation, self-esteem, and psychological resilience. Table 6 presents the correlation coefficients for these psychological variables.

**Table 6:** Correlation analysis of Chinese and Western classical music preferences on individual cognitive emotion regulation, self-esteem, and psychological resilience.

Variant	Emo	Esteem	Resilience
Western Classical Music Preferences	0.725 (0.000***)	0.687 (0.000***)	0.722 (0.000***)
Chinese Classical Music Preferences	0.634 (0.000***)	0.598 (0.000***)	0.617 (0.000***)

***, **, and * represent the 1, 5, and 10% significance levels, respectively.

The results indicate that Western classical music preferences exhibit greater correlation coefficients for cognitive emotion regulation, self-esteem, and psychological resilience compared to Chinese classical music preferences. Specifically, the correlation coefficients for Western classical music preferences are 0.725 for cognitive emotion regulation (*p* < 0.001), 0.687 for self-esteem (*p* < 0.001), and 0.722 for psychological resilience (*p* < 0.001). In comparison, the correlation coefficients for Chinese classical music preferences are 0.634 (*p* < 0.001), 0.598 (*p* < 0.001), and 0.617 (*p* < 0.001) for the same three variables.

These results suggest that both Western and Chinese classical music preferences have significant positive effects on cognitive emotion regulation, self-esteem, and psychological resilience. However, Western classical music preferences demonstrate a stronger effect. This finding is important for understanding the role of music preferences in promoting mental health and provides theoretical support for further exploration of how different music preferences influence mental well-being in different cultural contexts.

### 4.3 Regression analysis

Correlation analyses identified relationships between Chinese and Western classical music preferences and individuals’ cognitive emotion regulation, self-esteem, and psychological resilience.

To test these hypotheses, separate regression analyses were performed for each psychological variable. Cognitive emotion regulation, self-esteem, and psychological resilience were set as dependent variables, while age, sex, education level, marital status, area of residence, annual income (RMB), ethnicity, religion, and Chinese and Western music preferences were taken as independent variables. Six regression models were constructed, and the results are presented in Table 7.

**Table 7:** Effects of Chinese and Western classical music preferences on cognitive emotion regulation, self-esteem, and psychological resilience.

Variant	Emo		Esteem		Resilience	
	1	2	3	4	5	6
wcmp	0.609*** (25.846)		0.504*** (23.318)		0.66*** (25.734)	
ccmp		0.559*** (20.577)		0.455*** (18.445)		0.591*** (19.641)
age	−0.023 (−1.018)	−0.031 (−1.278)	−0.049** (−2.418)	−0.057** (−2.54)	−0.021 (−0.869)	−0.031 (−1.153)
gnd	−0.090 (−1.54)	−0.14** (−2.159)	−0.016 (−0.299)	−0.058 (−0.978)	−0.075 (−1.185)	−0.13* (−1.808)
edu	0.039 (1.625)	0.039 (1.446)	0.029 (1.309)	0.028 (1.162)	0.046* (1.754)	0.045 (1.514)
mrtl	0.035 (0.578)	0.138** (2.041)	0.053 (0.957)	0.137** (2.228)	0.038 (0.572)	0.147* (1.954)
area	−0.131** (−2.244)	−0.105 (−1.626)	−0.118** (−2.214)	−0.097 (−1.642)	−0.18*** (−2.848)	−0.152** (−2.117)
incm	0.067** (2.452)	0.1*** (3.272)	0.041 (1.625)	0.068** (2.452)	0.065** (2.160)	0.1*** (2.964)
ethn	−0.055 (−0.832)	−0.161** (−2.201)	0.123** (2.037)	0.035 (0.533)	−0.052 (−0.721)	−0.166** (−2.042)
relg	0.157** (2.49)	0.097 (1.39)	0.163*** (2.830)	0.115* (1.805)	0.141** (2.063)	0.079 (1.013)
F	78.63***	41.141	64.484***	41.141	77.464***	45.891
Adjustment of R^2^	0.537	0.375	0.487	0.375	0.533	0.401
Within-group coefficient difference *p*-value	0.0872*		0.0408**		0.0243**	

***, **, and * represent the 1, 5, and 10% significance levels, respectively. The Within-group coefficient difference *p*-value is calculated based on the estimated results of the test using the Seemingly Unrelated Regression model, and its result is roughly similar to that of the Chow test.

The regression analysis of cognitive emotion regulation, centered around Models 1 and 2, disclosed adjusted R^2^ values of 0.537 and 0.375, along with F-statistics of 78.63 and 41.141, respectively. Both of these attained statistical significance at the *p* < 0.001 threshold. This reveals that the predictor variables elucidated 53.7 and 37.5% of the variance in cognitive emotion regulation. In Model 1, the regression coefficient for Western classical music preferences stood at 0.609 (*t* = 25.846, *p* < 0.001). In Model 2, the regression coefficient for Chinese classical music preferences was 0.559 (*t* = 20.577, *p* < 0.001). The Within-group coefficient difference *p*-value of 0.0872* insinuated, to a certain degree, a marginally significant difference among the within-group coefficients, thus verifying Hypothesis H1a.

The regression analysis of self-esteem, focusing on Models 3 and 4, presented adjusted R^2^ values of 0.487 and 0.375, with *F*-statistics of 64.484 and 41.141, respectively. Both were significant at the *p* < 0.001 level. This signified that the predictor variables accounted for 48.7 and 37.5% of the variance in self-esteem. In Model 3, the regression coefficient for Western classical music preferences was 0.504 (*t* = 23.318, *p* < 0.001). In Model 4, the regression coefficient for Chinese classical music preferences was 0.455 (*t* = 18.445, *p* < 0.001). Moreover, the Within-group coefficient difference *p-*value of 0.0408** clearly demonstrated that there was a significant difference among the within-group coefficients, thereby confirming Hypothesis H2b.

The regression analysis of psychological resilience, centered on Models 5 and 6, revealed adjusted R^2^ values of 0.533 and 0.401, accompanied by *F*-statistics of 77.464 and 45.891, respectively. Both achieved statistical significance at the *p* < 0.001 mark. This fully manifested that the predictor variables could account for 53.3 and 40.1% of the variance in psychological resilience, respectively. In Model 5, the regression coefficient for Western classical music preferences was 0.66 (*t* = 25.734, *p* < 0.001). In Model 6, the regression coefficient for Chinese classical music preferences was 0.591 (*t* = 19.641, *p* < 0.001). Notably, the Within-group coefficient difference *p*-value of 0.0243** evidently indicated that there was a significant difference among the within-group coefficients, thereby providing robust support for Hypothesis H2c.

## 5 Discussion

From a cross-cultural comparative perspective, this study systematically investigated the differential impacts of preferences for Chinese and Western classical music on cognitive emotion regulation, self-esteem, and psychological resilience. Through the analysis of 604 valid samples, the study reveals the correlation between music preferences and mental health indicators, providing a theoretical basis for the practice of music therapy.

The results show that preferences for both Chinese and Western classical music are significantly and positively correlated with cognitive emotion regulation, self-esteem, and psychological resilience, thus verifying the core hypotheses H1a-H1c. With its unique artistic charm, classical music can exert a positive regulatory effect on an individual’s psychological state through various physiological, cognitive, and social mechanisms. These findings are consistent with previous research, further consolidating the understanding of music’s broad impact on mental health (Osborne et al., 2014; Pauwels et al., 2014; Peistaraite and Clark, 2020).

In further comparisons, we found that the preference for Western classical music had stronger effects than the preference for Chinese classical music in enhancing cognitive emotion regulation, self-esteem, and psychological resilience, which also confirmed hypotheses H2a, H2b, and H2c. This advantage of Western classical music may stem from its complex harmonic structure and rich emotional expression. Western music emphasizes harmonic complexity and polyphonic textures, utilizing a “tension-resolution” model in sonata form to evoke emotional catharsis through vertical chord progressions and to activate reward-related brain regions such as the ventral striatum. This may allow it to more effectively regulate emotions and enhance self-esteem and psychological resilience. In contrast, Chinese classical music is primarily based on monophonic melodies built on the pentatonic scale, emphasizing horizontal melodic contours and subtle changes in timbre. For instance, the breathy overtones and strategic pauses of the guqin are used to cultivate a meditative and introspective state, which aligns with the Daoist and Confucian concepts of natural harmony and emotional restraint. These cultural and musical structural differences render the psychological regulation of Chinese classical music more subtle and implicit.

However, the interpretation of this result must be approached with caution and framed within the “cultural positionality” of the participants. It must be emphasized that this finding arises from a specific cultural context—Chinese participants comparing their own cultural music with a foreign one—and should by no means be misinterpreted as a judgment that Chinese music is of lesser universal value than Western music. A more persuasive explanation is that cultural familiarity plays a key confounding role. In the context of globalization, the internet, and modern education systems, Chinese listeners’ exposure to and familiarity with Western classical theory and repertoire may have surpassed their in-depth knowledge of their own traditional music. This familiarity bias, rather than the inherent qualities of the music itself, is highly likely to be the primary reason for the observed difference in effects. Therefore, the findings of this study cannot be generalized to describe the functional role of Chinese music for all cultural groups.

## 6 Conclusion

This study explored the comprehensive effects of Chinese and Western classical music on adults’ cognitive emotion regulation, self-esteem, and psychological resilience, and examined the moderating role of individual factors. The findings indicate that classical music significantly enhances all measured psychological indicators, with the most pronounced impact on resilience. Specifically, compared to Chinese classical music, Western classical music demonstrated stronger effects on cognitive emotion regulation and self-esteem. Among the moderating factors, age and education level had a significant influence, whereas gender did not.

Based on these findings, this study holds important practical implications and policy value. On an applied level, it is recommended to integrate the structural properties of music with specific clinical needs. For example, future research could explore integrating the “tension-resolution” model of Western classical music into trauma-focused therapies to enhance emotional catharsis and resilience. At the public health policy level, efforts should move beyond general funding increases toward more targeted interventions, such as providing differentiated musical resources and guidance based on the cultural backgrounds and needs of different socioeconomic groups.

Although this study provides valuable insights, its limitations must be acknowledged. First, the study primarily relied on questionnaires and failed to directly measure and control for key variables such as cultural familiarity, personal music preferences, and listening frequency. Second, the cross-sectional design cannot establish causal relationships. Furthermore, the study’s interdisciplinary contextualization requires strengthening. This research only conducts comparisons within the domain of music and fails to make horizontal comparisons between the psychological effects of music and other types of cognitive or emotional stimuli (e.g., visual arts, natural landscapes), which limits our assessment of music’s uniqueness and relative effectiveness as an intervention tool. Therefore, future research should adopt longitudinal designs and expand the cultural diversity of samples to more deeply analyze the underlying mechanisms of music’s psychological benefits.

In summary, this study confirms the benefits of exposure to classical music for mental health and reveals the differential effects of different musical systems within a specific cultural context. The research underscores that the future direction should be a deeper integration of music education, clinical psychotherapy, and cultural studies. This interdisciplinary collaboration is essential for leveraging music more precisely and effectively to enhance individual and societal resilience and well-being.

## Data Availability

The raw data supporting the conclusions of this article will be made available by the authors, without undue reservation.
